# Level of Consciousness Is Dissociable from Electroencephalographic Measures of Cortical Connectivity, Slow Oscillations, and Complexity

**DOI:** 10.1523/JNEUROSCI.1910-19.2019

**Published:** 2020-01-15

**Authors:** Dinesh Pal, Duan Li, Jon G. Dean, Michael A. Brito, Tiecheng Liu, Anna M. Fryzel, Anthony G. Hudetz, George A. Mashour

**Affiliations:** ^1^Department of Anesthesiology, University of Michigan, Ann Arbor, Michigan 48109-5615,; ^2^Neuroscience Graduate Program, University of Michigan, Ann Arbor, Michigan 48109-2215,; ^3^Center for Consciousness Science, University of Michigan, Ann Arbor, Michigan 48109, and; ^4^Department of Molecular and Integrative Physiology, University of Michigan, Ann Arbor, Michigan 48109-5622

**Keywords:** anesthesia, cholinergic, consciousness, EEG, functional connectivity, noradrenergic

## Abstract

Leading neuroscientific theories posit a central role for the functional integration of cortical areas in conscious states. Considerable evidence supporting this hypothesis is based on network changes during anesthesia, but it is unclear whether these changes represent state-related (conscious vs unconscious) or drug-related (anesthetic vs no anesthetic) effects. We recently demonstrated that carbachol delivery to prefrontal cortex (PFC) restored wakefulness despite continuous administration of the general anesthetic sevoflurane. By contrast, carbachol delivery to parietal cortex, or noradrenaline delivery to either prefrontal or parietal cortices, failed to restore wakefulness. Thus, carbachol-induced reversal of sevoflurane anesthesia represents a unique state that combines wakefulness with clinically relevant anesthetic concentrations in the brain. To differentiate the state-related and drug-related associations of cortical connectivity and dynamics, we analyzed the electroencephalographic data gathered from adult male Sprague Dawley rats during the aforementioned experiments for changes in functional cortical gamma connectivity (25–155 Hz), slow oscillations (0.5–1 Hz), and complexity (<175 Hz). We show that higher gamma (85–155 Hz) connectivity is decreased (*p* ≤ 0.02) during sevoflurane anesthesia, an expected finding, but was not restored during wakefulness induced by carbachol delivery to PFC. Conversely, for rats in which wakefulness was not restored, the functional gamma connectivity remained reduced, but there was a significant decrease (*p* < 0.001) in the power of slow oscillations and increase (*p* < 0.001) in cortical complexity, which was similar to that observed during wakefulness induced after carbachol delivery to PFC. We conclude that the level of consciousness can be dissociated from cortical connectivity, oscillations, and dynamics.

**SIGNIFICANCE STATEMENT** Numerous theories of consciousness suggest that functional connectivity across the cortex is characteristic of the conscious state and is reduced during anesthesia. However, it is unknown whether the observed changes are state-related (conscious vs unconscious) or drug-related (drug vs no drug). We used a novel rat model in which cholinergic stimulation of PFC produced wakefulness despite continuous exposure to a general anesthetic. We demonstrate that, as expected, general anesthesia reduces connectivity. Surprisingly, the connectivity remains suppressed despite pharmacologically induced wakefulness in the presence of anesthetic, with restoration occurring only after the anesthetic is discontinued. Thus, whether an animal exhibits wakefulness or not can be dissociated from cortical connectivity, prompting a reevaluation of the role of connectivity in level of consciousness.

## Introduction

The biological basis of consciousness is considered to be among the most fundamental questions in science. A number of prominent theories of consciousness focus on the integration of neural information in cortical networks as measured via the strength or repertoire of functional brain connections (Dehaene and Changeux, 2011; Baars et al., 2013; Tononi et al., 2016; Carhart-Harris, 2018). Studies across multiple species and from multiple laboratories, including our own, suggest that (1) disruption of frontal-parietal functional connectivity (Lee et al., 2009, 2013; Ku et al., 2011; Schrouff et al., 2011; Boly et al., 2012; Hudetz, 2012; Jordan et al., 2013; Bonhomme et al., 2016; Pal et al., 2016; Ranft et al., 2016; Schroeder et al., 2016; Li et al., 2017; Bodart et al., 2018; Sanders et al., 2018a), (2) increase in spectral power of slow oscillations (Lewis et al., 2012; Ní Mhuircheartaigh et al., 2013; Purdon et al., 2013; Warnaby et al., 2017), and (3) reduced spatiotemporal complexity (Casali et al., 2013; Hudetz et al., 2015, 2016; Sarasso et al., 2015; Schartner et al., 2015; Li and Mashour, 2019) are correlates of anesthetic-induced unconsciousness. However, it is not clear whether the anesthetic-induced disruption in cortical connectivity, increase in the spectral power of slow oscillations, and reduction in cortical complexity correlate with unconsciousness (i.e., a state effect) or reflect the presence of anesthetic drugs in the brain (i.e., a drug effect). A primary reason for this gap in our understanding is that, normally, the presence of anesthetic drugs in the brain at clinically relevant concentrations is associated with the absence of wakefulness. Conversely, wakefulness normally occurs in the absence of anesthetic drugs or when these drugs reach subanesthetic concentrations in the brain. In a recent study (Pal et al., 2018), we demonstrated that cholinergic stimulation, via local carbachol delivery, of prefrontal cortex (PFC) in sevoflurane-anesthetized rat was sufficient to restore wakefulness despite the continuous presence of sevoflurane at concentrations (1.9%–2.4%) associated with surgical anesthesia. If preserved frontal-parietal connectivity, low power of slow oscillations, and/or high spatiotemporal complexity are indeed correlates of wakefulness, then these should be restored during carbachol-induced wakefulness despite the presence of anesthetic in the brain. Conversely, if disruption of frontal-parietal connectivity, enhanced power of slow oscillations, and/or reduced spatiotemporal complexity correlate with the presence of anesthetics in the brain, then pharmacological restoration of wakefulness in the presence of sevoflurane will not be associated with restoration to baseline wake levels. Interestingly, in our previous study (Pal et al., 2018), we also demonstrated that cholinergic stimulation of parietal cortex or the noradrenergic stimulation of either prefrontal or parietal cortices in sevoflurane-anesthetized rats activated the electroencephalogram (EEG) but failed to reverse general anesthesia and restore wakefulness, creating alternative models for understanding the state- and drug-related effects of general anesthetics.

In the current study, we analyzed the EEG datasets collected as part of these experiments (Pal et al., 2018) and demonstrate that (1) functional cortical gamma connectivity remains suppressed during ongoing sevoflurane exposure despite the concurrent induction of wakefulness following carbachol delivery into PFC; and (2) the changes in slow oscillations and cortical complexity correlate with changes in EEG activation rather than behavior. These findings suggest that the level of consciousness can be dissociated from EEG measures of connectivity and dynamics, prompting a reevaluation of the precise role in the mechanism and monitoring of consciousness.

## Materials and Methods

We used the intracranial EEG data from our recently published study (Pal et al., 2018) to analyze the changes in functional cortical gamma connectivity (25–155 Hz), spectral power of slow oscillations (0.5–1 Hz), and Lempel–Ziv complexity (<175 Hz), before, during, and after reverse dialysis delivery of carbachol and noradrenaline (NA) into the PFC and parietal cortex of sevoflurane-anesthetized rats. All experiments were conducted in adult male Sprague Dawley rats (300–350 g, Charles River Laboratories) maintained on a 12 h light/12 h dark cycle (lights on at 6:00 A.M.) with *ad libitum* food and water. The experiments were approved by the Institutional Animal Care and Use Committee (University of Michigan, Ann Arbor, Michigan) and were in compliance with the *Guide for the care and use of laboratory animals* (Ed 8, National Academies Press) as well as the ARRIVE guidelines. The EEG data were collected from five cortical sites: one frontal (from bregma: anterior 3.0 mm and mediolateral 2.5 mm), two parietal (from bregma: posterior 4.0 mm and mediolateral 2.5 mm), and two occipital (from bregma: posterior 8.0 mm and mediolateral 2.5 mm). One subset of rats was implanted with a microdialysis probe in PFC (from bregma: anterior 3.0 mm, mediolateral 0.5 mm, ventral 4.0 mm, and contralateral to the frontal EEG electrode) for microdialysis sample collection and local delivery of carbachol or NA, whereas the other subset was implanted with a microdialysis probe in parietal cortex (from bregma: posterior 3.6 mm, mediolateral 2.6 mm, ventral 2.0 mm, and at an angle of 40 degrees) for microdialysis sample collection and local delivery of carbachol or NA. All stereotaxic coordinates were based on the rat atlas by Paxinos and Watson (2007). A limitation of the conventional EEG recording montage as used in the previous study (Pal et al., 2018) is that, in the absence of high-density spatial data, it allows only temporal complexity analysis or the measurement of the diversity of the EEG signal only in the temporal domain. To overcome this limitation and examine the changes in spatiotemporal complexity, in the current study, we surgically prepared another group of rats (*n* = 7) for intracranial high-density EEG recording (30 cortical sites). To the best of our knowledge, this approach to sample EEG data from across the cortical surface in combination with microdialysis delivery and sample collection has not been reported previously. The procedures for the surgical implantation of electrodes to record EEG and the guide tubes for microdialysis delivery of carbachol or NA as well as the chromatographic quantification of local acetylcholine levels have been described in detail in our previous study (Pal et al., 2018), and the same procedures were adopted for the high-density EEG cohort in the current study.

### 

#### 

##### Experimental design.

**Figure 1.**
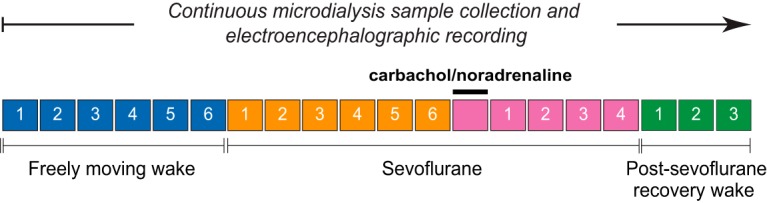
Schematic illustrating the experimental design. The EEG and microdialysis data were gathered simultaneously and without any interruption, but the dialysis samples were collected in 12.5 min bins, as illustrated by individual boxes in the schematic. Carbachol (5 mm) and NA (20 mm) were delivered during sevoflurane anesthesia using reverse dialysis while simultaneously collecting the dialysis fractions for quantification of the local acetylcholine levels (Pal et al., 2018). The same experimental plan was followed for the high-density EEG data and microdialysis sample collection in the current study.

The experimental design for the EEG data collection before, during, and after sevoflurane anesthesia along with microdialysis delivery of carbachol or NA into prefrontal and parietal cortices is illustrated in Figure 1. The detailed experimental procedures have been described in our previous study (Pal et al., 2018). In brief, on the day of the experiment, the rats were connected to the EEG recording cable, and a microdialysis probe being continuously perfused (2 μl/min using a CMA/400 syringe pump, CMA Microdialysis, Harvard Apparatus) with Ringer's solution (147 mm NaCl, 2.4 mm CaCl_2_, 4.0 mm KCl, 10 μm neostigmine; pH 6.0 ± 0.2), was lowered into either prefrontal (CMA/11, 1-mm-long cuprophane membrane, 0.24-mm-diameter, 6 kDa membrane cutoff) or parietal cortex (CMA/11, 2-mm-long cuprophane membrane, 0.24-mm-diameter, 6 kDa membrane cutoff) for carbachol or NA delivery and simultaneous collection of microdialysis samples. The EEG data were collected during baseline wake state for 75 min. To hold the behavioral state constant during the baseline condition, the rats were kept awake by introduction of novel objects and gentle tapping on the recording chamber. At the completion of 75 min of baseline wake recording, sevoflurane exposure (1.9%–2.4%) was started and titrated to maintain (1) loss of righting reflex, which is a widely accepted behavioral surrogate for anesthetic-induced unconsciousness in rats, and (2) high-amplitude, slow-wave EEG. The anesthetic exposure was continued for 75 min, after which either carbachol (5 mm) or NA (20 mm) was reverse dialyzed for 12.5 min into the PFC (*n* = 10 rats for carbachol and *n* = 8 rats for NA) or the parietal cortex (*n* = 11 rats each for carbachol and NA) while the rats were still inhaling the same concentration of sevoflurane anesthesia. At the end of carbachol or NA delivery, the EEG recordings continued under sevoflurane anesthesia for another 50 min. Thereafter, sevoflurane exposure was stopped and EEG data were collected for 37.5 min during the post-sevoflurane recovery wake state. The use of reverse dialysis delivery allowed us to restrict the spatial spread of carbachol or NA, thereby avoiding nonspecific effects associated with activation or inhibition of spatially distinct brain regions. Importantly, administration of carbachol and NA through reverse dialysis allowed us to simultaneously monitor the changes in local acetylcholine levels, which have been reported in our previous study (Pal et al., 2018). In the current study, the new cohort of male Sprague Dawley rats (*n* = 7) prepared for intracranial high-density EEG recordings was also implanted with a microdialysis probe in PFC for measuring acetylcholine levels during local reverse dialysis delivery of 5 mm carbachol. The following EEG segments, visually inspected and free of any artifacts, were selected for analysis: (1) baseline wake condition: last 300 s of the baseline wake state before exposure to sevoflurane anesthesia; (2) sevoflurane anesthesia: last 300 s of the sevoflurane exposure before pharmacological stimulation with carbachol or NA; (3) carbachol: 300 s from the point of first visible change in EEG after the start of carbachol delivery during sevoflurane anesthesia; (4) NA: 90–220 s from the point of first visible change in EEG after the start of NA delivery during sevoflurane anesthesia; and (5) post-sevoflurane recovery wake state: 300 s from the recovery of righting reflex, which occurred within 750 s after the cessation of sevoflurane anesthesia. The emergence time from sevoflurane anesthesia, as indicated by the return of righting reflex, was variable among rats, but all the rats recovered within 750 s of sevoflurane discontinuation. The epoch selection and the data analysis scheme for the high-density cohort were the same as that followed for the five-channel prefrontal-carbachol group described above and reported previously (Pal et al., 2018).

##### EEG recording.

Monopolar EEG signals were recorded with reference to a stainless-steel electrode over nasal sinus, as has been reported previously in studies from our (Borjigin et al., 2013; Pal et al., 2015, 2016) and other (Castro et al., 2013, 2014) laboratories. The signals were amplified (5000×) and bandpass filtered between 0.1 and 300 Hz using a Grass model 15 LT bipolar portable physiodata amplifier system (15A54 Quad Amplifier, Natus Neurology), and digitized at 1 kHz using MP150 data acquisition unit (Acq*knowledge* software version 4.1.1, Biopac Systems). The high-density EEG dataset was recorded on a Scout Grapevine Neural Processor (Ripple Neuro) using a bandpass filter of 0.1–300 Hz and sampling rate of 1 kHz. The raw EEG data were exported into MATLAB (version 2015a; The MathWorks) and downsampled to 500 Hz (resample.m function in MATLAB signal processing toolbox) for further analysis.

##### Corticocortical coherence analysis.

Corticocortical coherence was measured by the magnitude squared coherence method using the mscohere.m function in MATLAB signal processing toolbox (The MathWorks). The EEG data were segmented into nonoverlapped 10 s windows, which were further divided into 2 s subwindows with 80% overlap. Each subwindow was multiplied with a Hamming window, and the coherence was estimated from the cross-spectra and auto-spectra of the two EEG signals using Welch's averaged-modified periodogram method. The coherence for each window was estimated as a function of frequency (0.5–250 Hz) and between each pair of the five EEG channels. The mean global coherence was obtained by averaging the coherence for individual channel pairs for each animal in the following gamma frequency bands: low gamma (25–55 Hz), medium gamma (85–125 Hz), and high gamma (125–155 Hz). The choice of these frequency bands was based on our recent rat study in which we demonstrated high gamma (85–155 Hz) corticocortical coherence and frontal-parietal connectivity as a correlate of wakefulness that is disrupted during general anesthesia and sleep (Pal et al., 2016).

##### Frontal-parietal directed connectivity: normalized symbolic transfer entropy analysis.

Normalized symbolic transfer entropy is an information theoretic measure that is considered a surrogate for directed cortical communication. Our previous findings with normalized symbolic transfer entropy as a surrogate for frontal-parietal directed connectivity changes in human subjects (Lee et al., 2009, 2013; Ku et al., 2011) and rats (Borjigin et al., 2013; Pal et al., 2016; Li et al., 2017) have been supported by reports from other laboratories that have used methodologically different approaches (Boly et al., 2012; Jordan et al., 2013). In the current study, we analyzed the EEG data for changes in directed connectivity between ipsilateral frontal and parietal channels in the gamma frequency bandwidth: low gamma (25–55 Hz), medium gamma (85–125 Hz), and high gamma (125–155 Hz). We used a Butterworth filter of order 4 (butter.m and filtfilt.m, MATLAB signal processing toolbox) to filter the raw EEG data for the frequency bands of interest (i.e., low, medium, and high gamma), and segmented the filtered data into nonoverlapped 10 s windows. The calculation of normalized symbolic transfer entropy requires three parameters: embedding dimension, time delay, and prediction time. We fixed the embedding dimension at 3, and time delay at 5, 2, and 1, corresponding to low, medium, and high gamma, respectively. For each window, we searched the prediction time between 1 and 50 (corresponding to 2–100 ms) and selected the one that yielded maximum normalized symbolic transfer entropy in the frontal-to-parietal and parietal-to-frontal direction. These parameters for normalized symbolic transfer entropy have been used in our previous rat studies (Borjigin et al., 2013; Pal et al., 2016; Li et al., 2017).

##### Spectral power analysis.

The absolute power for the slow EEG oscillations was assessed in the temporal domain by applying a bandpass filter (0.5–1 Hz) to the EEG data using the eegfiltnew function in the EEGLAB toolbox (Delorme and Makeig, 2004). The power values were calculated for each nonoverlapped 10 s window, and the averaged power across all the windows and all the available channels was computed.

##### Temporal Lempel–Ziv complexity analysis.

Lempel–Ziv complexity computes the complexity of data with finite length sequences (Lempel and Ziv, 1976; Ziv and Lempel, 1977, 1978) and has been shown to be a valuable tool to investigate the neurophysiological changes associated with states of consciousness (Casali et al., 2013; Abásolo et al., 2015; Schartner et al., 2015, 2017a,b; Hudetz et al., 2016; Li and Mashour, 2019). Because of the limited number of EEG channels (Pal et al., 2018), we could not compute the spatial complexity and therefore focused on the temporal complexity in ipsilateral frontal and parietal channels. The EEG signals were detrended using local linear regression with a 10 s window at a 5 s overlap (locdetrend function in Chronux analysis software), and lowpass filtered at 175 Hz via Butterworth filter of order 5 (butter and filtfilt functions in MATLAB signal processing toolbox). The instantaneous amplitude was calculated from the Hilbert transform of the signal, which was then binarized using its mean value as the threshold for each channel (Schartner et al., 2017a). The binary sequence was segmented into nonoverlapped 10 s windows. For each of these 10 s windows, the Lempel–Ziv complexity algorithm searches for the instances of consecutive characters or “words” and counts the number of times a new word is encountered. To assess the degree to which the difference in the complexity across the states is not due to spectral changes, we generated surrogate data through phase randomization while preserving the spectral profiles of the signal (Schartner et al., 2015, 2017a), and normalized the original Lempel–Ziv complexity by the mean of the Lempel–Ziv complexity values from *N* = 50 surrogate time series. The resultant normalized Lempel–Ziv complexity values reflect complexity beyond the spectral changes, which were then averaged across all the windows as the estimate of the complexity at each studied state.

##### Spatiotemporal Lempel–Ziv complexity analysis.

To overcome the limitations of low channel count (five) and to reveal the complexity changes in terms of spatial diversity, we implanted a new cohort of rats (*n* = 7) in the current study for high-density EEG recordings. The spatial distribution of electrodes (30) covering the entire cortex allowed us to quantify spatiotemporal complexity, and in addition served as an important control group to ascertain the validity of the temporal complexity analysis conducted on five-channel EEG data. The EEG data in 3 of 7 rats in the high-density cohort were not useable because of excessive artifacts and noise levels. After excluding these rats and any bad channels, the remaining EEG signals (number of channels: 25–29) were detrended, lowpass filtered at 175 Hz, and divided into nonoverlapped 2 s windows after removing the common signal across channels. For each window, the instantaneous amplitude was estimated by applying the Hilbert transform (Schartner et al., 2015, 2017a), which was binarized using its mean value as the threshold for each channel. The data were then converted into a binary matrix, in which rows represent channels and columns represent time points. The complexity of the spatiotemporal matrix was assessed by Lempel–Ziv complexity (Casali et al., 2013; Li and Mashour, 2019), which searches the binary matrix, time point by time point, and counts the number of different spatial patterns across different time points. We then normalized the spatiotemporal complexity by the mean of those from the surrogate data through phase randomization, to examine whether the difference in the spatiotemporal complexity across the states is due to spectral changes.

##### Statistical analyses.

Statistical analyses were conducted in consultation with the Consulting for Statistics, Computing and Analytics Research unit at the University of Michigan (Ann Arbor, Michigan). The initial study (Pal et al., 2018) from which the EEG data were used in the current study was designed to have 80% power at α of 0.05. All statistical comparisons were conducted in a within-group design using the programming and statistical language R (version 3.6.0) (R Core Team, 2019). We used a linear mixed model with random intercept for each rat. This accounts for the correlations among observations of the same rat and allows unified reporting of all pairwise state comparisons. *Post hoc* pairwise tests were single-step corrected for multiple comparisons (Package *multicomp*). Models were fit with restricted maximum likelihood. *p* values of <0.05 were considered statistically significant. For clarity and readability, we have provided the *p* values in Results, whereas the mean, SD, *F* statistics, and effect sizes for all datasets and comparisons (except the acetylcholine dataset) have been provided in tabular format, as referenced in Results. The acetylcholine dataset has relatively fewer comparisons because of which we have reported the associated statistical values in the text in Results.

## Results

### Corticocortical gamma coherence does not correlate with level of consciousness

#### Carbachol delivery into prefrontal and parietal cortices

**Figure 2.**
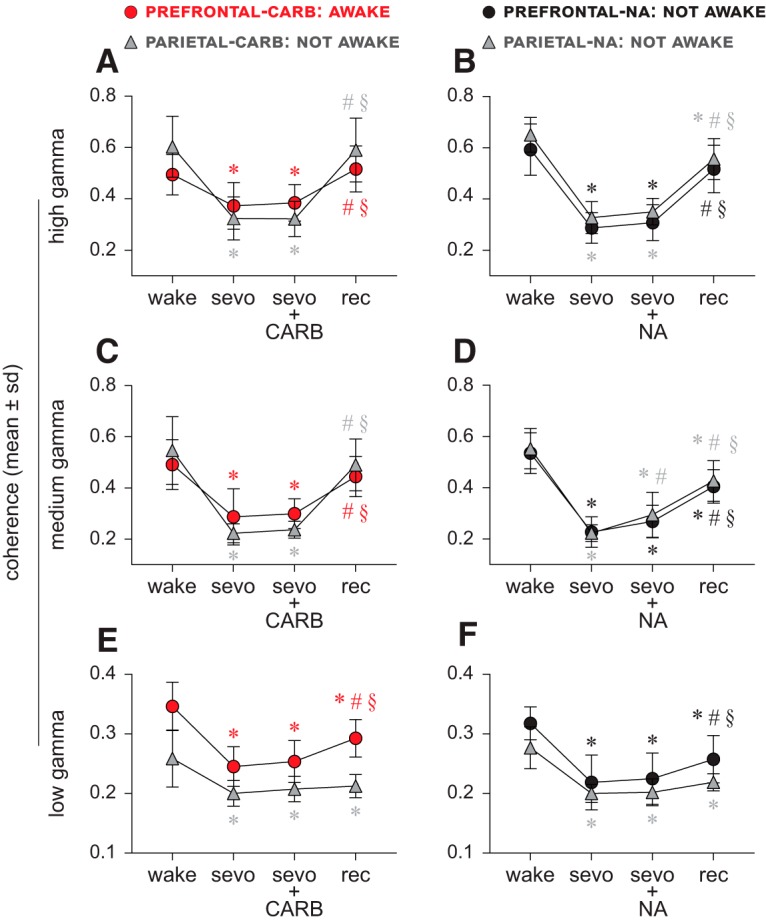
Corticocortical coherence does not correlate with level of consciousness. ***A–F***, Sevoflurane anesthesia (sevo) significantly reduced corticocortical coherence in high (125–155 Hz), medium (85–125 Hz), and low (25–55 Hz) gamma range. Cholinergic stimulation of PFC through reverse dialysis delivery of 5 mm carbachol (CARB) was reported to induce wakefulness in the presence of sevoflurane anesthesia (Pal et al., 2018) but failed to restore gamma coherence (high, medium, low) to baseline wake levels (***A***,***C***,***E***). Similar delivery of CARB into parietal cortex or 20 mm NA into prefrontal and parietal cortices during sevoflurane anesthesia did not restore wakefulness (Pal et al., 2018), and none of these interventions restored disrupted gamma coherence (***A–F***). The gamma coherence during the post-sevoflurane recovery wake epoch (rec) showed a trend toward return to baseline wake levels (***A–F***) but remained significantly low, except in the high and medium gamma bands for prefrontal- and parietal cortex-CARB (***A***) and prefrontal-NA (***B***) groups. A linear mixed model with random intercept for each rat was used for the statistical comparisons, and the *post hoc* pairwise tests were single-step corrected for multiple comparisons. The statistical comparisons are shown at *p* < 0.05. The mean, SD, exact *p* values, *F* statistics, and the effect sizes for each statistical comparison are provided in Tables 1 and 2. The significance symbols are color-coded to match the line-symbol plots. *Significant compared with baseline wake state. ^#^Significant compared with sevo. §Significant compared with CARB or NA during sevo.

Compared with the baseline wake state, sevoflurane anesthesia was characterized by a significant reduction (*p* ≤ 0.001) in the corticocortical coherence across the gamma bandwidth in both the prefrontal and parietal cortical groups (Fig. 2*A*,*C*,*E*; Table 1). Carbachol delivery into PFC of sevoflurane-anesthetized rats was shown to restore wakefulness despite the continuous presence of sevoflurane anesthesia (Pal et al., 2018). However, analysis of the EEG from the same epoch showed that, despite the restoration of wakefulness, the corticocortical gamma coherence (high, medium, low) was not statistically different from that observed during sevoflurane anesthesia (*p* ≥ 0.74), and remained significantly reduced (*p* ≤ 0.0017) compared with the baseline wake state (Fig. 2*A*,*C*,*E*; Table 1). Wakefulness was determined based on clear attempts at righting or recovery of the righting reflex. All rats in the prefrontal carbachol group showed such behavioral responses and 4 of 11 rats regained complete mobility. Detailed descriptions of behavioral and physiological changes, as well as a representative movie clip showing completely mobile animal after carbachol delivery, are provided in our previous publication (Pal et al., 2018). As opposed to the effect of carbachol in PFC, the reverse dialysis delivery of carbachol into parietal cortex of sevoflurane-anesthetized rat was ineffective in restoring wakefulness (Pal et al., 2018) and analysis of the EEG during the same epoch showed that coherence across the gamma bandwidth remained significantly low (*p* < 0.001) compared with the baseline wake state (Fig. 2*A*,*C*,*E*; Table 1); there was no significant difference in coherence in any of the gamma bands between the sevoflurane anesthesia epoch and the carbachol delivery epoch (*p* ≥ 0.88). The post-sevoflurane recovery wake state in the prefrontal and parietal groups was characterized by increase in coherence in high gamma (*p* ≤ 0.001 compared with sevoflurane, *p* < 0.001 compared with carbachol epochs) and medium gamma (*p* < 0.001 compared with sevoflurane, *p* < 0.001 compared with carbachol epochs) bands, which returned to the baseline wake levels (*p* ≥ 0.12) (Fig. 2*A*,*C*; Table 1). The low gamma coherence during post-sevoflurane recovery wake state in both prefrontal and parietal groups remained significantly below (*p* < 0.001) than that observed during the baseline wake state (Fig. 2*E*; Table 1), even though there was a significant increase in the PFC group compared with both sevoflurane (*p* < 0.001) and carbachol delivery (*p* < 0.001) epochs (Fig. 2*E*; Table 1).

**Table 1.** Corticocortical coherence before, during, and after sevoflurane administration and carbachol delivery into prefrontal and parietal cortices

	Low gamma				Medium gamma				High gamma			
	Wake	Sevo	CARB	Rec	Wake	Sevo	CARB	Rec	Wake	Sevo	CARB	Rec
PFC–carbachol												
Wake	0.35 (0.041)*^a^*	−0.10 (0.027)	−0.093 (0.032)	−0.054 (0.034)	0.49 (0.097)*^a^*	−0.20 (0.12)	−0.19 (0.093)	−0.047 (0.10)	0.49 (0.079)*^a^*	−0.12 (0.11)	−0.11 (0.085)	−0.022 (0.11)
Sevo	*p* < 0.001	0.25 (0.033)*^a^*	0.0084 (0.019)	0.048 (0.019)	*p* < 0.001	0.29 (0.11)*^a^*	0.012 (0.087)	0.16 (0.096)	*p* < 0.001	0.37 (0.091)*^a^*	0.012 (0.11)	0.14 (0.11)
CARB	*p* < 0.001	*p* = 0.74	0.25 (0.035)*^a^*	0.039 (0.021)	*p* < 0.001	*p* = 0.98	0.30 (0.058)*^a^*	0.15 (0.036)	*p* = 0.0017	*p* = 0.98	0.38 (0.071)*^a^*	0.13 (0.038)
Rec	*p* < 0.001	*p* < 0.001	*p* < 0.001	0.29 (0.032)*^a^*	*p* = 0.39	*p* < 0.001	*p* < 0.001	0.45 (0.078)*^a^*	*p* = 0.89	*p* < 0.001	*p* < 0.001	0.52 (0.090)*^a^*
	*F*_(3,27)_ = 63; *p* < 0.001				*F*_(3,27)_ = 25; *p* < 0.001				*F*_(3,27)_ = 12; *p* < 0.001			
Parietal cortex–carbachol												
Wake	0.26 (0.048)*^a^*	−0.059 (0.038)	−0.051 (0.042)	−0.046 (0.046)	0.55 (0.13)*^a^*	−0.32 (0.11)	−0.31 (0.11)	−0.056 (0.078)	0.60 (0.12)*^a^*	−0.28 (0.087)	−0.28 (0.10)	−0.013 (0.10)
Sevo	*p* < 0.001	0.20 (0.021)*^a^*	0.0072 (0.021)	0.012 (0.013)	*p* < 0.001	0.22 (0.037)*^a^*	0.014 (0.028)	0.27 (0.075)	*p* < 0.001	0.32 (0.084)*^a^*	−0.0018 (0.061)	0.27 (0.13)
CARB	*p* < 0.001	*p* = 0.88	0.21 (0.021)*^a^*	0.0051 (0.018)	*p* < 0.001	*p* = 0.95	0.24 (0.033)*^a^*	0.25 (0.079)	*p* < 0.001	*p* = 1	0.32 (0.069)*^a^*	0.27 (0.11)
Rec	*p* < 0.001	*p* = 0.59	*p* = 0.96	0.21 (0.019)*^a^*	*p* = 0.12	*p* < 0.001	*p* < 0.001	0.49 (0.10)*^a^*	*p* = 0.98	*p* < 0.001	*p* < 0.001	0.59 (0.13)*^a^*
	*F*_(3,30)_ = 15; *p* < 0.001				*F*_(3,30)_ = 88; *p* < 0.001				*F*_(3,30)_ = 54; *p* < 0.001			

*^a^*Mean (±SD) for each state is shown along the diagonal of each section.

The cells above the mean (±SD) values show the effect sizes as the difference of the means (±SD). Sevo, Sevoflurane administration; CARB, carbachol delivered during sevo; Rec, post-sevoflurane recovery wake.

#### NA delivery into prefrontal and parietal cortices

Sevoflurane anesthesia produced a significant reduction (*p* < 0.001) in the corticocortical coherence across the gamma bandwidth (Fig. 2*B*,*D*,*F*; Table 2). Reverse dialysis delivery of NA into either prefrontal or parietal cortex during sevoflurane anesthesia was not observed to produce any signs of wakefulness (Pal et al., 2018). During the same epoch (NA delivery), the corticocortical coherence in high gamma (*p* < 0.001) and medium gamma (*p* < 0.001) bands in both prefrontal and parietal groups remained significantly reduced compared with the baseline wake state (Fig. 2*B*,*D*; Table 2), even though there was a significant increase (*p* = 0.021 compared with sevoflurane) in medium gamma coherence in the parietal cortex group. The post-sevoflurane recovery wake state in both prefrontal and parietal groups was also characterized by a significant increase in high and medium gamma coherence (*p* < 0.001) compared with both sevoflurane and NA epochs (Fig. 2*B*,*D*; Table 2). Although the increase in high gamma coherence during post-sevoflurane recovery period in the prefrontal group reached the baseline levels and was not statistically different from the wake state (*p* = 0.14), the high gamma coherence in parietal group remained significantly lower than the baseline levels (*p* = 0.0036). Similarly, the medium gamma coherence during the post-sevoflurane recovery period, in both prefrontal and parietal groups, remained significantly lower (*p* < 0.001) than the baseline wake levels. The low gamma coherence during post-sevoflurane recovery wake state in the prefrontal group increased compared with sevoflurane (*p* < 0.001) and NA delivery (*p* < 0.001) epochs but still remained significantly low (*p* < 0.001) compared with baseline wake state (Fig. 2*F*; Table 2). In the parietal group, the low gamma coherence during the post-sevoflurane epoch remained significantly low (*p* < 0.001) compared with the baseline wake levels and was not significantly different from either sevoflurane (*p* = 0.11) or NA (*p* = 0.17) epochs (Fig. 2*F*; Table 2).

**Table 2.** Corticocortical coherence before, during, and after sevoflurane administration and NA delivery into prefrontal and parietal cortices

	Low gamma				Medium gamma				High gamma			
	Wake	Sevo	NA	Rec	Wake	Sevo	NA	Rec	Wake	Sevo	NA	Rec
PFC–NA												
Wake	0.32 (0.028)*^a^*	−0.099 (0.023)	−0.093 (0.023)	−0.060 (0.024)	0.54 (0.079)*^a^*	−0.31 (0.053)	−0.27 (0.080)	−0.13 (0.074)	0.59 (0.10)*^a^*	−0.31 (0.12)	−0.29 (0.13)	−0.076 (0.11)
Sevo	*p* < 0.001	0.22 (0.046)*^a^*	0.0063 (0.014)	0.039 (0.029)	*p* < 0.001	0.23 (0.059)*^a^*	0.041 (0.036)	0.18 (0.061)	*p* < 0.001	0.29 (0.060)*^a^*	0.020 (0.035)	0.23 (0.096)
NA	*p* < 0.001	*p* = 0.87	0.23 (0.043)*^a^*	0.033 (0.025)	*p* < 0.001	*p* < 0.26	0.27 (0.063)*^a^*	0.14 (0.068)	*p* < 0.001	*p* = 0.94	0.31 (0.070)*^a^*	0.21 (0.089)
Rec	*p* < 0.001	*p* < 0.001	*p* < 0.001	0.26 (0.040)*^a^*	*p* < 0.001	*p* < 0.001	*p* < 0.001	0.41 (0.066)*^a^*	*p* = 0.14	*p* < 0.001	*p* < 0.001	0.52 (0.093)*^a^*
	*F*_(3,21)_ = 61; *p* < 0.001				*F*_(3,21)_ = 78; *p* < 0.001				*F*_(3,21)_ = 36; *p* < 0.001			
Parietal cortex–NA												
Wake	0.28 (0.035)*^a^*	−0.077 (0.032)	−0.075 (0.036)	−0.058 (0.029)	0.55 (0.079)*^a^*	−0.33 (0.068)	−0.26 (0.082)	−0.13 (0.11)	0.65 (0.067)*^a^*	−0.32 (0.068)	−0.30 (0.10)	−0.095 (0.12)
Sevo	*p* < 0.001	0.20 (0.015)*^a^*	0.0019 (0.024)	0.019 (0.018)	*p* < 0.001	0.22 (0.033)*^a^*	0.072 0.083	0.21 (0.083)	*p* < 0.001	0.33 (0.062)*^a^*	0.023 (0.064)	0.23 (0.11)
NA	*p* < 0.001	*p* = 1	0.20 (0.22)*^a^*	0.017 (0.021)	*p* < 0.001	*p* = 0.021	0.29 (0.088)*^a^*	0.13 (0.065)	*p* < 0.001	*p* = 0.84	0.35 (0.052)*^a^*	0.21 (0.079)
Rec	*p* < 0.001	*p* = 0.11	*p* = 0.17	0.22 (0.014)*^a^*	*p* < 0.001	*p* < 0.001	*p* < 0.001	0.43 (0.079)*^a^*	*p* = 0.0036	*p* < 0.001	*p* < 0.001	0.56 (0.080)*^a^*
	*F*_(3,30)_ = 38; *p* < 0.001				*F*_(3,30)_ = 69; *p* < 0.001				*F*_(3,30)_ = 65; *p* < 0.001			

*^a^*Mean (±SD) for each state is shown along the diagonal of each section.

The cells above the mean (±SD) values show the effect sizes as the difference of the means (±SD). Sevo, Sevoflurane administration; NA, NA delivered during sevo; Rec, post-sevoflurane recovery wake.

### Directed cortical gamma connectivity does not correlate with level of consciousness

#### Carbachol delivery into prefrontal and parietal cortices

**Figure 3.**
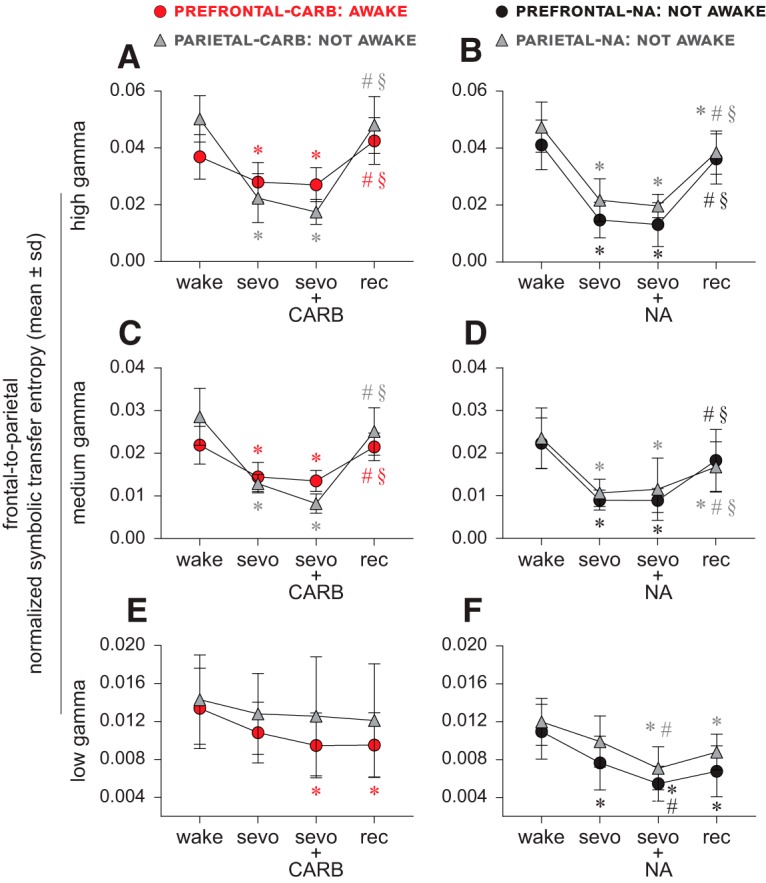
Frontal-to-parietal directed gamma connectivity does not correlate with level of consciousness. ***A–D***, Frontal-to-parietal connectivity in the high (125–155 Hz) and medium (85–125 Hz) gamma bands was significantly reduced during sevoflurane anesthesia (sevo). Neither the restoration of wakefulness through 5 mm carbachol (CARB) delivery to PFC nor the absence of wakefulness after CARB delivery to parietal cortex or 20 mm NA delivery to prefrontal and parietal cortices, as reported previously (Pal et al., 2018), had any significant effect on the high and medium gamma frontal-to-parietal connectivity, which remained disrupted and was not significantly different from that observed during sevoflurane anesthesia alone (***A–D***). The frontal-to-parietal connectivity during the post-sevoflurane recovery wake epoch (rec) returned to baseline wake levels (wake) for high and medium gamma bands for all conditions, except in the parietal cortex-NA group (***B***,***D***), where it remained significantly lower compared with wake. The connectivity changes in low gamma bandwidth were highly variable between groups (***E***,***F***). A linear mixed model with random intercept for each rat was used for the statistical comparisons, and the *post hoc* pairwise tests were single-step corrected for multiple comparisons. The statistical comparisons are shown at *p* < 0.05. The mean, SD, exact *p* values, *F* statistics, and the effect sizes for each statistical comparison are provided in Tables 3 and 5. The significance symbols are color-coded to match the line-symbol plots. *Significant compared with wake. ^#^Significant compared with sevo. §Significant compared with CARB or NA during sevo.

**Figure 4.**
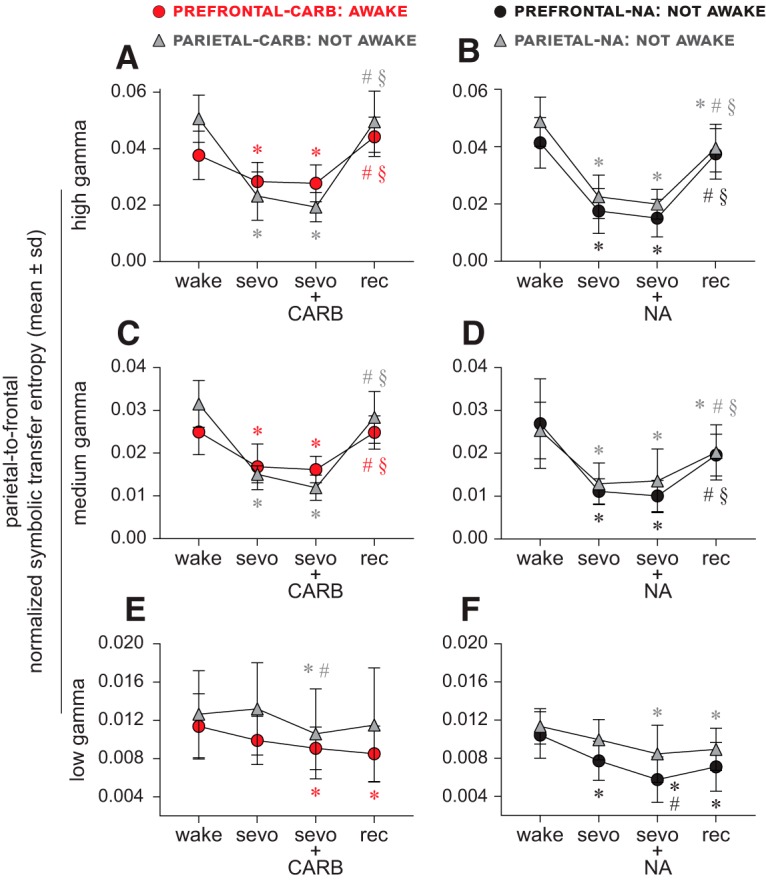
Parietal-to-frontal directed gamma connectivity does not correlate with level of consciousness. ***A–D***, Parietal-to-frontal connectivity in the high (125–155 Hz) and medium (85–125 Hz) gamma bands was significantly reduced during sevoflurane anesthesia (sevo). Neither the restoration of wakefulness through 5 mm carbachol (CARB) delivery to PFC nor the absence of wakefulness after CARB into parietal cortex or 20 mm NA delivery to prefrontal and parietal cortices, as reported previously (Pal et al., 2018), had any significant effect on the high and medium gamma parietal-to-frontal connectivity, which remained disrupted and was not significantly different from that observed during sevoflurane anesthesia alone (***A–D***). The parietal-to-frontal connectivity during the post-sevoflurane recovery wake epoch (rec) returned to baseline wake (wake) levels for high and medium gamma bands for all conditions, except in the parietal cortex-NA group (***A***) where it remained significantly lower compared with wake. The connectivity changes in low gamma bandwidth were highly variable between groups (***E***,***F***). A linear mixed model with random intercept for each rat was used for the statistical comparisons, and the *post hoc* pairwise tests were single-step corrected for multiple comparisons. The statistical comparisons are shown at *p* < 0.05. The mean, SD, exact *p* values, *F* statistics, and the effect sizes for each statistical comparison are provided in Tables 4 and 6. The significance symbols are color-coded to match the line-symbol plots. *Significant compared with wake. ^#^Significant compared with sevo. §Significant compared with CARB or NA during sevo.

Compared with the baseline wake state, sevoflurane anesthesia was characterized by a significant suppression of bidirectional (frontal-to-parietal and parietal-to-frontal) connectivity in the high and medium gamma bands (*p* ≤ 0.020) in both prefrontal and parietal groups (Figs. 3*A*,*C*, 4*A*,*C*; Tables 3, 4). The delivery of carbachol into prefrontal, but not parietal cortex, was earlier shown to restore wakefulness in the presence of sevoflurane anesthesia (Pal et al., 2018). However, bidirectional frontal-parietal gamma connectivity was not restored after delivery of carbachol into either PFC (restored wakefulness) or parietal cortex (no signs of wakefulness). High and medium gamma connectivity remained significantly below the baseline wake levels (high gamma: *p* < 0.0074; medium gamma: *p* < 0.001) and was not significantly different from that observed during sevoflurane anesthesia (*p* ≥ 0.070) (Figs. 3*A*,*C*, 4*A*,*C*; Tables 3, 4). In both the prefrontal and parietal groups, the post-sevoflurane recovery wake state was characterized by a significant increase in high gamma and medium gamma bidirectional frontal-parietal connectivity (*p* < 0.001 compared with both sevoflurane and carbachol epochs) (Figs. 3*A*,*C*, 4*A*,*C*; Tables 3, 4); the connectivity levels in both the groups returned to baseline wake levels (*p* ≥ 0.1). The bidirectional frontal-parietal connectivity in low gamma bandwidth showed widely variable effects (Figs. 3*E*, 4*E*; Tables 3, 4).

**Table 3.** Frontal-to-parietal connectivity before, during, and after sevoflurane administration and carbachol delivery into prefrontal and parietal cortices

	Low gamma				Medium gamma				High gamma			
	Wake	Sevo	CARB	Rec	Wake	Sevo	CARB	Rec	Wake	Sevo	CARB	Rec
PFC–carbachol												
Wake	0.013 (0.0042)*^a^*	−0.0026 (0.0030)	−0.0039 (0.0040)	−0.0039 (0.0031)	0.022 (0.0045)*^a^*	−0.0075 (0.0058)	−0.0084 (0.0054)	−44e-5 (0.0044)	0.037 (0.0079)*^a^*	−0.0089 (0.0082)	−0.010 (0.010)	0.0056 (0.010)
Sevo	*p* = 0.044	0.011 (0.0032)*^a^*	−0.0014 (0.0020)	−0.0013 (0.0026)	*p* < 0.001	0.014 (0.0034)*^a^*	−93e-5 (0.0041)	0.0071 (0.0050)	*p* = 0.020	0.028 (0.0069)*^a^*	−93e-5 (0.011)	0.015 (0.012)
CARB	*p* < 0.001	*p* = 0.51	0.0095 (0.0034)*^a^*	38e-6 (0.0035)	*p* < 0.001	*p* = 0.92	0.014 (0.0025)*^a^*	0.0080 (0.0023)	*p* = 0.0074	*p* = 1	0.027 (0.0060)*^a^*	0.015 (0.0067)
Rec	*p* < 0.001	*p* = 0.54	*p* = 1	0.010 (0.0034)*^a^*	*p* = 1	*p* < 0.001	*p* < 0.001	0.022 (0.0032)*^a^*	*p* = 0.27	*p* < 0.001	*p* < 0.001	0.043 (0.0082)*^a^*
	*F*_(3,27)_ = 7.0; *p* = 0.0012				*F*_(3,27)_ = 19; *p* < 0.001				*F*_(3,27)_ = 12; *p* < 0.001			
Parietal cortex–carbachol												
Wake	0.014 (0.0047)*^a^*	−0.0015 (0.0028)	−0.0017 (0.0041)	−0.0022 (0.0032)	0.029 (0.0067)*^a^*	−0.016 (0.0069)	−0.020 (0.0081)	−0.0034 (0.0075)	0.050 (0.0082)*^a^*	−0.028 (0.010)	−0.033 (0.0095)	−0.0022 (0.011)
Sevo	*p* = 0.41	0.013 (0.0043)*^a^*	−24e-5 (0.0034)	−69e-5 (0.0023)	*p* < 0.001	0.013 (0.0022)*^a^*	−0.0047 (0.0027)	0.012 (0.0060)	*p* < 0.001	0.022 (0.0086)*^a^*	−0.0049 0.0065	0.026 (0.014)
CARB	*p* = 0.28	*p* = 1	0.013 (0.0063)*^a^*	−45e-5 (0.0032)	*p* < 0.001	*p* = 0.070	0.0082 (0.0023)*^a^*	0.017 (0.0056)	*p* < 0.001	*p* = 0.43	0.017 (0.0044)*^a^*	0.031 (0.011)
Rec	*p* = 0.11	*p* = 0.89	*p* = 0.97	0.012 (0.0060)*^a^*	*p* = 0.28	*p* < 0.001	*p* < 0.001	0.025 (0.0056)*^a^*	*p* = 0.91	*p* < 0.001	*p* < 0.001	0.048 (0.010)*^a^*
	*F*_(3,30)_ = 1.9; *p* = 0.15				*F*_(3,30)_ = 51; *p* < 0.001				*F*_(3,30)_ = 56; *p* < 0.001			

*^a^*Mean (±SD) for each state is shown along the diagonal of each section.

The cells above the mean (±SD) values show the effect sizes as the difference of the means (±SD). Sevo, Sevoflurane administration; CARB, carbachol delivered during sevo; Rec, post-sevoflurane recovery wake.

**Table 4.** Parietal-to-frontal connectivity before, during, and after sevoflurane administration and carbachol delivery into prefrontal and parietal cortices

	Low gamma				Medium gamma				High gamma			
	Wake	Sevo	CARB	Rec	Wake	Sevo	CARB	Rec	Wake	Sevo	CARB	Rec
PFC–carbachol												
Wake	0.011 (0.0034)*^a^*	−0.0015 (0.023)	−0.0023 (0.0029)	−0.0029 (0.0019)	0.025 (0.0053)*^a^*	−0.0081 (0.0068)	−0.0088 (0.0058)	−11e-5 (0.0054)	0.038 (0.0086)*^a^*	−0.0093 (0.0087)	−0.0099 (0.0090)	0.0065 (0.0091)
Sevo	*p* = 0.18	0.0099 (0.0025)*^a^*	−83e-5 (0.0018)	−0.0014 (0.0022)	*p* < 0.001	0.017 (0.0053)*^a^*	−66e-5 (0.0054)	0.0080 (0.0064)	*p* = 0.0077	0.028 (0.0068)*^a^*	−58e-5 (0.011)	0.016 (0.011)
CARB	*p* = 0.0077	*p* = 0.66	0.0091 (0.0022)*^a^*	−58e-5 (0.0025)	*p* < 0.001	*p* = 0.98	0.016 (0.0031)*^a^*	0.0087 (0.0020)	*p* = 0.0038	*p* = 1	0.028 (0.0065)*^a^*	0.017 (0.0046)
Rec	*p* < 0.001	*p* = 0.21	*p* = 0.85	0.0085 (0.0029)*^a^*	*p* = 1	*p* < 0.001	*p* < 0.001	0.025 (0.0039)*^a^*	*p* = 0.11	*p* < 0.001	*p* < 0.001	0.044 (0.0070)*^a^*
	*F*_(3,27)_ = 6.0; *p* = 0.0029				*F*_(3,27)_ = 16; *p* < 0.001				*F*_(3,27)_ = 15; *p* < 0.001			
Parietal cortex–carbachol												
Wake	0.013 (0.0045)*^a^*	55e-5 (0.0016)	−0.0021 (0.0025)	−0.0011 (0.0030)	0.031 (0.0052)*^a^*	−0.016 (0.0041)	−0.019 (0.0064)	−0.0030 (0.0060)	0.051 (0.0084)*^a^*	−0.028 (0.011)	−0.031 (0.011)	−0.0011 (0.012)
Sevo	*p* = 0.88	0.013 (0.0048)*^a^*	−0.0026 (0.0024)	−0.0017 (0.0025)	*p* < 0.001	0.015 (0.0020)*^a^*	−0.0030 (0.0035)	0.013 (0.0053)	*p* < 0.001	0.023 (0.0086)*^a^*	−0.0039 (0.0071)	0.026 (0.016)
CARB	*p* = 0.031	*p* = 0.0028	0.011 (0.0047)*^a^*	92e-5 (0.0028)	*p* < 0.001	*p* = 0.26	0.012 (0.0028)*^a^*	0.016 (0.0060)	*p* < 0.001	*p* = 0.71	0.019 (0.0052)*^a^*	0.030 (0.013)
Rec	*p* = 0.43	*p* = 0.11	*p* = 0.61	0.012 (0.0060)*^a^*	*p* = 0.23	*p* < 0.001	*p* < 0.001	0.028 (0.0058)*^a^*	*p* = 1	*p* < 0.001	*p* < 0.001	0.050 (0.011)*^a^*
	*F*_(3,30)_ = 4.9; *p* = 0.0072				*F*_(3,30)_ = 71; *p* < 0.001				*F*_(3,30)_ = 43; *p* < 0.001			

*^a^*Mean (±SD) for each state is shown along the diagonal of each section.

The cells above the mean (±SD) values show the effect sizes as the difference of the means (±SD). Sevo, Sevoflurane administration; CARB, carbachol delivered during sevo; Rec, post-sevoflurane recovery wake.

#### NA delivery into prefrontal and parietal cortices

Sevoflurane anesthesia was characterized by a significant suppression of bidirectional (frontal-to-parietal and parietal-to-frontal) connectivity in the high and medium gamma bands (*p* < 0.001) in both prefrontal and parietal groups (Figs. 3*B*,*D*, 4*B*,*D*; Tables 5, 6). NA delivery into the prefrontal or parietal cortex of sevoflurane-anesthetized rats did not produce any signs of wakefulness (Pal et al., 2018), and the analysis of the concomitant EEG showed no significant change in the high gamma or medium gamma bidirectional frontal-parietal connectivity (*p* ≥ 0.85 compared with sevoflurane anesthesia), which remained significantly below the baseline wake levels (*p* < 0.001) (Figs. 3*B*,*D*, 4*B*,*D*; Tables 5, 6). The post-sevoflurane recovery wake epoch in both prefrontal and parietal groups was characterized by a significant increase in bidirectional frontal-parietal connectivity in both high gamma (*p* < 0.001 compared with sevoflurane anesthesia and NA epochs) and medium gamma (*p* ≤ 0.029 compared with sevoflurane anesthesia, *p* ≤ 0.0094 compared with NA) bands (Figs. 3*B*,*D*, 4*B*,*D*; Tables 5, 6); the high and medium gamma bidirectional connectivity in the prefrontal group returned to baseline wake levels (*p* ≥ 0.077) but in the parietal cortex group it remained significantly below (*p* ≤ 0.019) the baseline wake levels (Figs. 3*B*,*D*, 4*B*,*D*; Tables 5, 6). The bidirectional frontal-parietal connectivity in low gamma bandwidth showed widely variable effects (Figs. 3*F*, 4*F*; Tables 5, 6).

**Table 5.** Frontal-to-parietal connectivity before, during, and after sevoflurane administration and NA delivery into prefrontal and parietal cortices

	Low gamma				Medium gamma				High gamma			
	Wake	Sevo	NA	Rec	Wake	Sevo	NA	Rec	Wake	Sevo	NA	Rec
PFC–NA												
Wake	0.011 (0.0029)*^a^*	−0.0033 (0.0014)	−0.0055 (0.0019)	−0.0042 (0.0014)	0.022 (0.0060)*^a^*	−0.013 (0.0062)	−0.013 (0.0081)	−0.0040 (0.0087)	0.041 (0.0087)*^a^*	−0.026 (0.014)	−0.028 (0.014)	−0.0049 (0.011)
Sevo	*p* < 0.001	0.0076 (0.0028)*^a^*	−0.0022 (0.0013)	−87e-5 (0.0016)	*p* < 0.001	0.0090 (0.0024)*^a^*	−83e-6 (0.0024)	0.0093 (0.0075)	*p* < 0.001	0.015 (0.0063)*^a^*	−0.0016 (0.0038)	0.021 (0.0084)
NA	*p* < 0.001	*p* < 0.001	0.0055 (0.0018)*^a^*	0.0013 (0.0014)	*p* < 0.001	*p* = 1	0.0089 (0.0028)*^a^*	0.0094 (0.0082)	*p* < 0.001	*p* = 0.97	0.013 (0.0077)*^a^*	0.023 (0.0074)
Rec	*p* < 0.001	*p* = 0.36	*p* = 0.066	0.0068 (0.0027)*^a^*	*p* = 0.38	*p* = 0.0012	*p* = 0.0010	0.018 (0.0073)*^a^*	*p* = 0.54	*p* < 0.001	*p* < 0.001	0.036 (0.0088)*^a^*
	*F*_(3,21)_ = 38; *p* < 0.001				*F*_(3,28)_ = 14; *p* < 0.001				*F*_(3,21)_ = 31; *p* < 0.001			
Parietal cortex–NA												
Wake	0.012 (0.0025)*^a^*	−0.0021 (0.0033)	−0.0049 (0.0029)	−0.0032 (0.0022)	0.024 (0.0071)*^a^*	−0.013 (0.0058)	−0.012 (0.0060)	−0.0068 (0.0058)	0.047 (0.0088)*^a^*	−0.026 (0.011)	−0.028 (0.010)	−0.0089 (0.013)
Sevo	*p* = 0.058	0.0099 (0.0027)*^a^*	−0.0028 (0.0034)	−0.0011 (0.0029)	*p* < 0.001	0.011 (0.0032)*^a^*	88e-5 (0.0062)	0.0061 (0.0052)	*p* < 0.001	0.022 (0.0075)*^a^*	−0.0021 (0.0059)	0.017 (0.011)
NA	*p* < 0.001	*p* = 0.0035	0.0071 (0.0023)*^a^*	0.0017 (0.0012)	*p* < 0.001	*p* = 0.96	0.012 (0.0073)*^a^*	0.0052 (0.0040)	*p* < 0.001	*p* = 0.90	0.020 (0.0041)*^a^*	0.019 (0.0070)
Rec	*p* < 0.001	*p* = 0.54	*p* = 0.16	0.0088 (0.0019)*^a^*	*p* < 0.001	*p* = 0.0014	*p* = 0.0091	0.017 (0.0059)*^a^*	*p* = 0.016	*p* < 0.001	*p* < 0.001	0.039 (0.0076)*^a^*
	*F*_(3,30)_ = 12; *p* < 0.001				*F*_(3,30)_ = 25; *p* < 0.001				*F*_(3,30)_ = 39; *p* < 0.001			

*^a^*Mean (±SD) for each state is shown along the diagonal of each section.

The cells above the mean (±SD) values show the effect sizes as the difference of the means (±SD). Sevo, Sevoflurane administration; NA, NA delivered during sevo; Rec, post-sevoflurane recovery wake.

**Table 6.** Parietal-to-frontal connectivity before, during, and after sevoflurane administration and NA delivery into prefrontal and parietal cortices

	Low gamma				Medium gamma				High gamma			
	Wake	Sevo	NA	Rec	Wake	Sevo	NA	Rec	Wake	Sevo	NA	Rec
PFC–NA												
Wake	0.010 (0.0024)*^a^*	−0.0027 (0.0020)	−0.0047 (0.0026)	−0.0033 (0.0019)	0.027 (0.010)*^a^*	−0.016 (0.010)	−0.017 (0.012)	−0.0073 (0.011)	0.041 (0.0088)*^a^*	−0.024 (0.012)	−0.026 (0.013)	−0.0038 (0.011)
Sevo	*p* < 0.001	0.0077 (0.0021)*^a^*	−0.0020 (0.0011)	−63e-5 (0.0017)	*p* < 0.001	0.011 (0.0029)*^a^*	−0.0011 (0.0022)	0.0085 (0.0055)	*p* < 0.001	0.018 (0.0078)*^a^*	−0.0025 (0.0054)	0.020 (0.011)
NA	*p* < 0.001	*p* = 0.018	0.0058 (0.0024)*^a^*	0.0013 (0.0019)	*p* < 0.001	*p* = 0.99	0.010 (0.0036)*^a^*	0.0096 (0.0063)	*p* < 0.001	*p* = 0.90	0.015 (0.0066)*^a^*	0.022 (0.0082)
Rec	*p* < 0.001	*p* = 0.79	*p* = 0.19	0.0071 (0.0026)*^a^*	*p* = 0.077	*p* = 0.029	*p* = 0.0094	0.020 (0.0049)*^a^*	*p* = 0.72	*p* < 0.001	*p* < 0.001	0.038 (0.0088)*^a^*
	*F*_(3,21)_ = 17; *p* < 0.001				*F*_(3,21)_ = 13; *p* < 0.001				*F*_(3,21)_ = 28; *p* < 0.001			
Parietal cortex–NA												
Wake	0.011 (0.0019)*^a^*	−0.0014 (0.0024)	−0.0029 (0.0039)	−0.0024 (0.0020)	0.025 (0.0066)*^a^*	−0.012 (0.0047)	−0.012 (0.0057)	−0.0051 (0.0059)	0.049 (0.0086)*^a^*	−0.026 (0.0095)	−0.029 (0.012)	−0.0092 (0.014)
Sevo	*p* = 0.39	0.0099 (0.0021)*^a^*	−0.0015 (0.0031)	−0.0010 (0.0026)	*p* < 0.001	0.013 (0.0049)*^a^*	69e-5 (0.0062)	0.0074 (0.0054)	*p* < 0.001	0.023 (0.0076)*^a^*	−0.0026 (0.0070)	0.017 (0.011)
NA	*p* = 0.0060	*p* = 0.33	0.0085 (0.0030)*^a^*	48e-5 (0.0032)	*p* < 0.001	*p* = 0.97	0.014 (0.0074)*^a^*	0.0067 (0.0032)	*p* < 0.001	*p* = 0.85	0.020 (0.0052)*^a^*	0.020 (0.0079)
Rec	*p* = 0.032	*p* = 0.66	*p* = 0.95	0.0089 (0.0022)*^a^*	*p* = 0.0071	*p* < 0.001	*p* < 0.001	0.020 (0.0064)*^a^*	*p* = 0.019	*p* < 0.001	*p* < 0.001	0.040 (0.0083)*^a^*
	*F*_(3,30)_ = 4.2; *p* = 0.014				*F*_(3,30)_ = 28; *p* < 0.001				*F*_(3,30)_ = 38; *p* < 0.001			

*^a^*Mean (±SD) for each state is shown along the diagonal of each section.

The cells above the mean (±SD) values show the effect sizes as the difference of the means (±SD). Sevo, Sevoflurane administration; NA, NA delivered during sevo; Rec, post-sevoflurane recovery wake.

### Spectral power in slow oscillations does not correlate with level of consciousness

#### Carbachol delivery into prefrontal and parietal cortices

**Figure 5.**
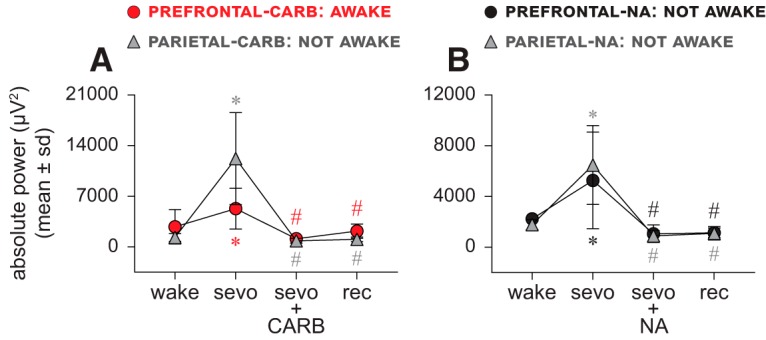
Spectral power of slow oscillations does not correlate with level of consciousness. ***A***, ***B***, Spectral power of slow oscillations (0.5–1 Hz) showed a significant increase during sevoflurane anesthesia (sevo). The restoration of wakefulness through 5 mm carbachol (CARB) delivery to PFC as well as the absence of wakefulness after CARB delivery to parietal cortex or 20 mm NA delivery to prefrontal and parietal cortices were reported to be accompanied with EEG activation (Pal et al., 2018), which produced a significant decrease (compared with sevo) in spectral power regardless of the presence or absence of wakefulness (***A***, ***B***). The spectral power during post-sevoflurane recovery wake (rec) epoch remained significantly lower than sevo and was not significantly different from CARB or NA or the baseline wake state (wake) (***A***, ***B***). A linear mixed model with random intercept for each rat was used for the statistical comparisons, and the *post hoc* pairwise tests were single-step corrected for multiple comparisons. The statistical comparisons are shown at *p* < 0.05. The mean, SD, exact *p* values, *F* statistics, and the effect sizes for each statistical comparison are provided in Table 7. The significance symbols are color-coded to match the line-symbol plots. *Significant compared with wake. ^#^Significant compared with sevo.

As expected, compared with the baseline wake state, sevoflurane anesthesia was associated with a significant increase (*p* ≤ 0.02) in spectral power in slow oscillations (Fig. 5*A*; Table 7). In our previous report (Pal et al., 2018), we showed that the delivery of carbachol into PFC of sevoflurane-anesthetized rats could restore wakefulness and produce EEG activation, while carbachol into parietal cortex produced only EEG activation (Pal et al., 2018). Analysis of the carbachol epoch showed that regardless of the presence (stimulation of PFC) or absence (stimulation of parietal cortex) of wakefulness, the EEG activation was accompanied by a significant decrease in slow oscillations power compared with the sevoflurane epoch (*p* ≤ 0.001 for carbachol in the PFC, *p* < 0.001 for carbachol in the parietal cortex) (Fig. 5*A*; Table 7). The post-sevoflurane recovery wake period in both the prefrontal and parietal groups was also characterized by decrease in the spectral power in slow oscillations that was significantly lower than that observed during sevoflurane anesthesia (*p* = 0.0018 for carbachol in the PFC, *p* < 0.001 for carbachol in the parietal cortex). For both the prefrontal and parietal cortical groups, there was no significant difference (*p* ≥ 0.23) in the spectral power between baseline wake state, carbachol-induced EEG activation, and the post-sevoflurane recovery wake period (Fig. 5*A*; Table 7).

**Table 7.** Absolute power of slow oscillations before, during, and after sevoflurane administration, and carbachol and NA delivery into prefrontal and parietal cortices

PFC-carbachol					PFC-NA				
	Wake	Sevo	CARB	Rec		Wake	Sevo	NA	Rec
Wake	2785 (2376)*^a^*	2506 (4309)	−1654 (2476)	−608 (2557)	Wake	2225 (419)*^a^*	3040 (3746)	−1171 (637)	−1087 (470)
Sevo	*p* = 0.021	5291 (2824)*^a^*	−4160 (3074)	−3113 (3576)	Sevo	*p* = 0.0051	5265 (3811)*^a^*	−4211 (3549)	−4127 (3519)
CARB	*p* = 0.23	*p* < 0.001	1131 (722)*^a^*	1047 (1285)	NA	*p* = 0.58	*p* < 0.001	1054 (692)*^a^*	84 (695)
Rec	*p* = 0.90	*p* = 0.0018	*p* = 0.62	2178 (986)*^a^*	Rec	*p* = 0.63	*p* < 0.001	*p* = 1	1138 (371)*^a^*
	*F*_(3,32)_ = 8.3; *p* < 0.001					*F*_(3,3)_ = 9.3; *p* = 0.069			

Parietal cortex–carbachol					Parietal cortex–NA				
	Wake	Sevo	CARB	Rec		Wake	Sevo	NA	Rec
Wake	1303 (533)*^a^*	10,900 (6044)	−458 (585)	−264 (393)	Wake	1772 (381)*^a^*	4708 (3273)	−890 (604)	−684 (769)
Sevo	*p* < 0.001	12,203 (6362)*^a^*	−11,358 6232)	−11,163 (6251)	Sevo	*p* < 0.001	6480 (3110)*^a^*	−5597 (3131)	−5392 (3134)
CARB	*p* = 0.99	*p* < 0.001	845 (337)*^a^*	194 (493)	NA	*p* = 0.56	*p* < 0.001	883 (419)*^a^*	205 (773)
Rec	*p* = 1	*p* < 0.001	*p* = 1	1040 (328)*^a^*	Rec	*p* = 0.75	*p* < 0.001	*p* = 0.99	1088 (530)*^a^*
	*F*_(3,72)_ = 36; *p* < 0.001					*F*_(3,44)_ = 30; *p* < 0.001			

*^a^*Mean (±SD) for each state is shown along the diagonal of each section.

The cells above the mean (±SD) values show the effect sizes as the difference of the means (±SD). Sevo, Sevoflurane administration; CARB, carbachol delivered during sevo; NA, NA delivered during sevo; Rec, post-sevoflurane recovery wake.

#### NA delivery into prefrontal and parietal cortices

Sevoflurane anesthesia produced a significant increase (*p* ≤ 0.0051) in the spectral power in slow oscillations in both the prefrontal and parietal cortical groups (Fig. 5*B*; Table 7). NA in prefrontal or parietal cortex did not restore wakefulness during ongoing sevoflurane administration, but it was shown to produce EEG activation (Pal et al., 2018). These findings are consistent with a previous study that demonstrated EEG activation without behavioral arousal in propofol-anesthetized rats after systemic treatment with a noradrenergic reuptake blocker (Kenny et al., 2015). The NA epoch, characterized by EEG activation, was also marked by a significant decrease in the spectral power in slow oscillations in both the prefrontal and parietal groups (*p* < 0.001 compared with sevoflurane anesthesia) (Fig. 5*B*; Table 7). The power in slow oscillations in the post-sevoflurane recovery wake period in both prefrontal and parietal groups was significantly reduced (*p* < 0.001) compared with sevoflurane anesthesia epoch (Fig. 5*B*; Table 7). For both the prefrontal and parietal cortical groups, there was no significant difference (*p* ≥ 0.56) in the spectral power between the post-sevoflurane recovery wake period, the NA-induced EEG activation, and the baseline wake state (Fig. 5*B*; Table 7).

### Electroencephalographic temporal Lempel–Ziv complexity does not correlate with level of consciousness

#### Carbachol delivery into prefrontal and parietal cortices

**Figure 6.**
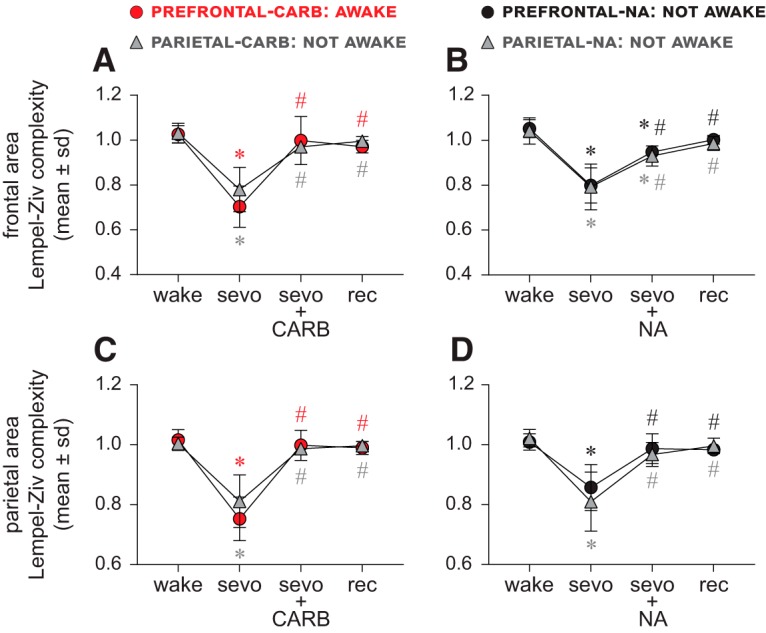
Electroencephalographic temporal Lempel–Ziv complexity does not correlate with level of consciousness. Sevoflurane anesthesia (sevo) was characterized by a significant reduction in Lempel–Ziv complexity in frontal (***A***, ***B***) and parietal areas (***C***, ***D***). The restoration of wakefulness through 5 mm carbachol (CARB) delivery to PFC as well as the absence of wakefulness after CARB delivery to parietal cortex or 20 mm NA delivery to prefrontal and parietal cortices was reported to be accompanied by EEG activation (Pal et al., 2018), which reversed the sevoflurane-induced decrease in complexity. The increase was significant (compared with sevo) for all groups and was not different from baseline wake state (wake), except for prefrontal- and parietal cortex-NA groups (***B–D***), where it remained significantly lower compared with wake. The complexity during post-sevoflurane recovery wake (rec) epoch remained significantly higher than sevo and was not significantly different from CARB or NA epochs or wake (***A–D***). The plotted values for complexity were obtained after normalizing raw temporal complexity data with phase-randomized surrogate time series and show complexity changes beyond that explained by only spectral changes. A linear mixed model with random intercept for each rat was used for the statistical comparisons, and the *post hoc* pairwise tests were single-step corrected for multiple comparisons. The statistical comparisons are shown at *p* < 0.05. The mean, SD, exact *p* values, *F* statistics, and the effect sizes for each statistical comparison are provided in Tables 8 and 9. The significance symbols are color-coded to match the line-symbol plots. *Significant compared with baseline wake state. ^#^Significant compared with sevo.

The temporal complexity was measured over the frontal and parietal areas. As compared with the baseline wake state, sevoflurane anesthesia was characterized by a significant reduction (*p* < 0.001) in the temporal complexity (Fig. 6*A*,*C*; Table 8). Carbachol delivery into PFC (restoration of wakefulness) or parietal cortex (no behavioral arousal) during sevoflurane anesthesia produced EEG activation, which was characterized by increase in temporal complexity (*p* < 0.001 compared with sevoflurane anesthesia) (Fig. 6*A*,*C*; Table 8). In both the prefrontal and parietal groups, the post-sevoflurane recovery wake period was characterized by high temporal complexity in frontal (Fig. 6*A*; Table 8) and parietal areas (Fig. 6*C*; Table 8) (*p* < 0.001 compared with sevoflurane anesthesia), which was not significantly different (*p* ≥ 0.28) from that observed during the baseline wake state and the carbachol delivery epoch (Fig. 6*A*,*C*; Table 8).

**Table 8.** Temporal Lempel–Ziv complexity in frontal and parietal areas before, during, and after sevoflurane administration and carbachol delivery into prefrontal and parietal cortices

	PFC–carbachol				Parietal cortex–carbachol			
	Wake	Sevo	CARB	Rec	Wake	Sevo	CARB	Rec
Frontal area								
Wake	1.0 (0.0390)*^a^*	−0.32 (0.086)	−0.028 (0.11)	−0.055 (0.049)	1.0 (0.043)*^a^*	−0.25 (0.092)	−0.062 (0.046)	−0.036 (0.037)
Sevo	*p* < 0.001	0.70 (0.092)*^a^*	0.29 (0.10)	0.27 (0.11)	*p* < 0.001	0.78 (0.099)*^a^*	0.19 (0.11)	0.22 0.10
CARB	*p* = 0.80	*p* < 0.001	1.0 (0.11)*^a^*	−0.027 (0.11)	*p* = 0.039	*p* < 0.001	0.97 (0.024)*^a^*	0.026 (0.027)
Rec	*p* = 0.28	*p* < 0.001	*p* = 0.81	0.97 (0.028)*^a^*	*p* = 0.41	*p* < 0.001	*p* = 0.69	1.0 (0.020)*^a^*
	*F*_(3,27)_ = 48; *p* < 0.001				*F*_(3,30)_ = 48; *p* < 0.001			
Parietal area								
Wake	1.0 (0.035)*^a^*	−0.26 (0.078)	−0.017 (0.060)	−0.026 (0.042)	1.0 (0.021)*^a^*	−0.19 (0.077)	−0.017 (0.030)	−0.0073 (0.026)
Sevo	*p* < 0.001	0.75 (0.073)*^a^*	0.25 (0.084)	0.24 (0.080)	*p* < 0.001	0.81 (0.089)*^a^*	0.18 (0.095)	0.19 (0.089)
CARB	*p* = 0.85	*p* < 0.001	1.0 (0.050)*^a^*	−0.0084 (0.057)	*p* = 0.81	*p* < 0.001	0.99 (0.019)*^a^*	0.0098 (0.016)
Rec	*p* = 0.63	*p* < 0.001	*p* = 0.98	0.99 (0.022)*^a^*	*p* = 0.98	*p* < 0.001	*p* = 0.96	1.0 (0.014)*^a^*
	*F*_(3,27)_ = 66; *p* < 0.001				*F*_(3,30)_ = 46; *p* < 0.001			

*^a^*Mean (±SD) for each state is shown along the diagonal of each section.

The cells above the mean (±SD) values show the effect sizes as the difference of the means (±SD). Sevo, Sevoflurane administration; CARB, carbachol delivered during sevo; Rec, post-sevoflurane recovery wake.

#### NA delivery into prefrontal and parietal cortices

Compared with the baseline wake state, the temporal complexity was significantly reduced during sevoflurane anesthesia (*p* < 0.001) (Fig. 6*B*,*D*; Table 9). NA delivery into PFC or parietal cortex of sevoflurane anesthetized rats did not produce any signs of wakefulness, but it did produce EEG activation (Pal et al., 2018). Analysis of the NA epoch in both the prefrontal and parietal cortical groups showed a significant increase in the temporal complexity over the frontal (*p* < 0.001) (Fig. 6*B*; Table 9) and parietal (*p* < 0.001) (Fig. 6*D*; Table 9) areas, compared with sevoflurane anesthesia; despite the increase, the temporal complexity in the frontal area (Fig. 6*B*; Table 9) remained significantly lower than that observed during the baseline wake levels (*p* < 0.001), whereas for the parietal area (Fig. 6*D*; Table 9) it was comparable with baseline wake state (*p* = 0.12). The temporal complexity in the frontal and parietal areas during the post-sevoflurane recovery wake period remained significantly high compared with sevoflurane anesthesia (*p* < 0.001) (Fig. 6*B*,*D*; Table 9) but was not significantly different from that observed during baseline wake state (*p* ≥ 0.2) and the NA delivery epoch (*p* ≥ 0.089) (Fig. 6*B*,*D*; Table 9).

**Table 9.** Temporal Lempel–Ziv complexity in frontal and parietal areas before, during, and after sevoflurane administration and NA delivery into prefrontal and parietal cortices

	PFC–NA				Parietal cortex–NA			
	Wake	Sevo	NA	Rec	Wake	Sevo	NA	Rec
Frontal area								
Wake	1.1 (0.040)*^a^*	−0.25 (0.077)	−0.10 (0.058)	−0.049 (0.036)	1.0 (0.058)*^a^*	−0.25 (0.13)	−0.11 (0.080)	−0.056 (0.070)
Sevo	*p* < 0.001	0.80 (0.078)*^a^*	0.14 (0.099)	0.20 (0.078)	*p* < 0.001	0.79 (0.10)*^a^*	0.14 (0.097)	0.19 (0.12)
NA	*p* < 0.001	*p* < 0.001	0.95 (0.026)*^a^*	0.055 (0.038)	*p* < 0.001	*p* < 0.001	0.93 (0.045)*^a^*	0.055 (0.069)
Rec	*p* = 0.15	*p* < 0.001	*p* = 0.089	1.0 (0.019)*^a^*	*p* = 0.18	*p* < 0.001	*p* = 0.19	0.99 (0.031)*^a^*
	*F*_(3,28)_ = 44; *p* < 0.001				*F*_(3,40)_ = 30; *p* < 0.001			
Parietal area								
Wake	1.0 (0.027)*^a^*	−0.15 (0.087)	−0.022 (0.066)	−0.026 (0.033)	1.0 (0.030)*^a^*	−0.21 (0.11)	−0.054 (0.051)	−0.025 (0.047)
Sevo	*p* < 0.001	0.86 (0.077)*^a^*	0.13 (0.068)	0.13 (0.072)	*p* < 0.001	0.81 (0.099)*^a^*	0.16 (0.089)	0.19 (0.11)
NA	*p* = 0.76	*p* < 0.001	0.99 (0.050)*^a^*	−0.0035 (0.042)	*p* = 0.12	*p* < 0.001	0.97 (0.040)*^a^*	0.029 (0.052)
Rec	*p* = 0.67	*p* < 0.001	*p* = 1	0.98 (0.011)*^a^*	*p* = 0.72	*p* < 0.001	*p* = 0.64	1.0 (0.026)*^a^*
	*F*_(3,21)_ = 19; *p* < 0.001				*F*_(3,40)_ = 31; *p* < 0.001			

*^a^*Mean (±SD) for each state is shown along the diagonal of each section.

The cells above the mean (±SD) values show the effect sizes as the difference of the means (±SD). Sevo, Sevoflurane administration; NA, NA delivered during sevo; Rec, post-sevoflurane recovery wake.

### Electroencephalographic spatiotemporal Lempel–Ziv complexity does not correlate with level of consciousness

Compared with the baseline wake state, there was a significant decrease (*p* < 0.001) in spatiotemporal complexity during sevoflurane anesthesia (Table 10). Reverse dialysis delivery of 5 mm carbachol into PFC of these high-density cohort rats (under constant sevoflurane anesthesia) restored wakefulness (defined by attempts or recovery of righting), produced EEG activation, and caused a significant increase in the spatiotemporal complexity (*p* < 0.001) compared with sevoflurane anesthesia; there was no significant difference compared with the baseline wake state (*p* = 0.58) (Table 10). The spatiotemporal complexity during the post-sevoflurane recovery wake epoch was significantly higher compared with sevoflurane anesthesia (*p* < 0.001) but was not significantly different from either baseline wake state (*p* = 0.95) or carbachol delivery epoch (*p* = 0.27) (Table 10). These changes in spatiotemporal complexity (Table 10) mirrored the changes in temporal complexity over frontal and parietal areas, as described earlier (Fig. 6*A–D*). Of note, we also measured changes in local acetylcholine levels before, during, and after sevoflurane anesthesia, as well as carbachol delivery to PFC in these rats. Statistical comparison using linear mixed model showed a significant effect on the acetylcholine levels across conditions (*F*_(3,9)_ = 8.2, *p* = 0.006). Compared with the baseline wake state, sevoflurane anesthesia was characterized by an 84% decrease in acetylcholine levels [mean ± SD (CI): 0.078 ± 0.043 (0.036–0.12) for sevoflurane vs 0.49 ± 0.35 (0.15–0.84) for wake state, *p* = 0.58], which is comparable with that reported in our previous publication (Pal et al., 2018). Carbachol delivery to PFC during sevoflurane anesthesia produced a highly significant increase in the local acetylcholine levels [mean ± SD (CI): 1.57 ± 0.92 (0.66–2.47), *p* < 0.001 compared with sevoflurane anesthesia], which remained significantly elevated during post-sevoflurane recovery period [mean ± SD (CI): 1.13 ± 0.87 (0.29–1.99), *p* = 0.007 compared with sevoflurane]. Overall, these changes in prefrontal acetylcholine levels after sevoflurane administration and carbachol delivery, both in the direction of change and magnitude, are consistent with the data reported in our previous study (Pal et al., 2018).

**Table 10.** Spatiotemporal Lempel–Ziv complexity before, during, and after sevoflurane administration and carbachol delivery into PFC

	Prefrontal–carbachol			
	Wake	Sevo	CARB	Rec
Wake	0.92 (0.018)*^a^*	−0.16 (0.024)	−0.017 (0.020)	0.0072 (0.014)
Sevo	*p* < 0.001	0.76 (0.033)*^a^*	0.14 (0.042)	0.17 (0.019)
CARB	*p* = 0.58	*p* < 0.001	0.90 (0.031)*^a^*	0.024 (0.033)
Rec	*p* = 0.95	*p* < 0.001	*p* = 0.27	0.92 (0.022)*^a^*
	*F*_(3,9)_ = 69; *p* < 0.001			

*^a^*Mean (±SD) for each state is shown along the diagonal of each section.

The cells above the mean (±SD) values show the effect sizes as the difference of the means (±SD). Sevo, Sevoflurane administration; CARB, carbachol delivered during sevo; Rec, post-sevoflurane recovery wake.

## Discussion

The divergent effects of cholinergic stimulation on the prefrontal and parietal cortices enabled the discovery that the level of consciousness can be dissociated from EEG measures of cortical functional connectivity and cortical dynamics (Table 11). A number of major theories of consciousness are grounded in the requirement for functional, directed, or effective connectivity across the cortex in the conscious state (Alkire et al., 2008; Dehaene and Changeux, 2011; Baars et al., 2013; Tononi et al., 2016). Indeed, these theories have made successful predictions regarding the reduction in connectivity strength or repertoire empirically observed during sleep, general anesthesia induced by distinct drugs, and pathological states of unconsciousness (Laureys et al., 1999; Massimini et al., 2005; Lee et al., 2009, 2013; Ferrarelli et al., 2010; Boly et al., 2011, 2012; Cimenser et al., 2011; Ku et al., 2011; Changeux, 2012; Lewis et al., 2012; Rosanova et al., 2012; Casali et al., 2013; Jordan et al., 2013; Sarasso et al., 2015; Hudetz and Mashour, 2016; Ranft et al., 2016; Schroeder et al., 2016; Li et al., 2017; Bodart et al., 2018; Sanders et al., 2018a; Uhrig et al., 2018; Hemmings et al., 2019). However, it has been unclear whether the reduction of functional or cortical connectivity played a causal role in the state transition, was associated more closely with loss of higher cognition rather than consciousness per se, or was an epiphenomenon. By dissociating level of consciousness from large-scale cortical connectivity, our findings prompt a reevaluation of the role of these connectivity measures in distinguishing states of consciousness and unconsciousness. Similarly, strategies for clinical monitoring of consciousness based on connectivity patterns (Ferrarelli et al., 2010; Ku et al., 2011; Rosanova et al., 2012; Jordan et al., 2013; Lee et al., 2013) should be reconsidered. Importantly, our findings apply only to the level of consciousness, which can be defined by objective observations, such as wakeful behavior. It is possible that disruptions of large-scale cortical connectivity critically impair the phenomenological contents of consciousness, conscious access, or cognitive processing (Mashour and Hudetz, 2017). Furthermore, because of our experimental design requiring a closed, air-tight chamber for continuous sevoflurane exposure, we could not test whether the absence of behavioral arousal or wake state after pharmacological manipulation of the cortex constitutes a truly unresponsive condition. Our inferences are based on the observations of spontaneous behaviors and speak only to the spontaneous changes in level of arousal. Our recent studies in human volunteers (Li et al., 2019) and surgical patients (Vlisides et al., 2019) reported dynamic changes in connectivity patterns under deep anesthesia, but these connectivity patterns were not sufficient to reliably distinguish the changes in the level of consciousness. Although supportive of our results in the current study, it is important to note that these recent studies in humans were restricted to bandwidths below low gamma frequency and entailed long periods of a stable and deep anesthetic plane. A previous study in rats demonstrated that the emergence from isoflurane anesthesia was characterized by multistep discreet fluctuations in spectral power as opposed to a discrete binary process or continuous path to recovery (Hudson et al., 2014). Similar studies in rats with long duration of anesthetics can further clarify whether the connectivity patterns, in particular in gamma frequency, also demonstrate a dynamic evolution.

**Table 11.** Summary of changes in cortical dynamics in the presence and absence of wakefulness during continuous sevoflurane administration and pharmacological manipulations

	Wakefulness*^a^*	No signs of wakefulness*^b^*
Cortical functional connectivity	Low	Low
Cortical slow oscillation power	Low	Low
Cortical complexity	High	High

*^a^*Carbachol delivery to PFC.

*^b^*Carbachol delivery to parietal, or NA delivery to prefrontal and parietal, cortices.

In both clinical and preclinical settings, the presence of increased spectral power of slow oscillations (0.5–1 Hz) is considered a signature of unconsciousness (Brown et al., 2010; Lewis et al., 2012; Ní Mhuircheartaigh et al., 2013; Purdon et al., 2013; Warnaby et al., 2017), whereas the appearance of a low-amplitude fast EEG, along with the dissipation of slow oscillations, correlates with the return of consciousness. In the current study, we did indeed observe the appearance of high-amplitude slow EEG and increase in spectral power of slow oscillations during sevoflurane anesthesia. However, the increased spectral power of slow oscillations was dissipated not only in association with restoration of wakefulness and accompanying EEG activation, an expected finding, but also in the cohorts that showed only EEG activation without any signs of wakefulness. These findings demonstrate that dissipation of slow oscillations correlates with an activated EEG rather than behavioral arousal, per se. Furthermore, the known occurrence of low-amplitude fast EEG, a pattern typically associated with wakefulness, during rapid eye movement sleep (Hobson, 2009), the occurrence of high-amplitude slow-wave EEG during wakefulness in patients with Angelman syndrome (Sidorov et al., 2017), and the experimental induction of high-amplitude slow-wave EEG in freely moving rats through systemic atropine administration (Qiu et al., 2015) all show various instances of dissociation between EEG patterns and level of consciousness. Similar reports from human subjects under general anesthesia further support these findings across species and states of unconsciousness. For instance, using the isolated forearm technique, Gaskell et al. (2017) demonstrated that the presence of frontal α-δ pattern in the EEG, typically considered to be a marker of anesthetic-induced unconsciousness, cannot reliably discriminate between behavioral responsiveness and unresponsiveness in certain patients under anesthesia, a conclusion that was supported by another patient report (Sanders et al., 2018b). Conversely, ketamine is known to produce fast EEG signatures that, unlike other commonly used general anesthetics, such as propofol or sevoflurane, are not reliably tracked by anesthesia monitors (Maksimow et al., 2006). Our data are consistent with these past findings by demonstrating that slow oscillations can be dissociated from level of consciousness.

Electroencephalographic complexity has been demonstrated to be high during wakefulness as well as in states with high phenomenological contents, such as rapid eye movement sleep (Abásolo et al., 2015; Schartner et al., 2017b) and the psychedelic experience (Tagliazucchi et al., 2014; Schartner et al., 2017a; Li and Mashour, 2019). On the other hand, complexity has been reported to be low during the states with reduced level of arousal, such as non-rapid eye movement sleep (Casali et al., 2013; Abásolo et al., 2015; Schartner et al., 2017b) and anesthesia (Casali et al., 2013; Hudetz et al., 2015, 2016; Sarasso et al., 2015). Based on these earlier reports, we expected that EEG complexity would increase after restoration of wakefulness, as achieved by prefrontal cholinergic stimulation, while remaining at a reduced level in the cohorts that failed to demonstrate restoration of wakefulness. Contrary to our expectation, temporal complexity, as measured through Lempel–Ziv algorithm, increased regardless of the presence or absence of wakefulness and instead correlated with EEG activation. In our earlier study (Pal et al., 2018), we showed that the restoration of wakefulness following carbachol-mediated cholinergic stimulation of PFC was accompanied by an approximately fivefold increase in local acetylcholine levels. More limited increases in local acetylcholine levels were also observed after NA delivery to PFC as well as carbachol or NA delivery to parietal cortex. In the current study, the changes in spatiotemporal Lempel–Ziv complexity also showed a positive relationship with prefrontal cholinergic tone and EEG activation, which aligns with the changes observed in temporal Lempel–Ziv complexity. Furthermore, the increase in complexity has been reported in states, such as rapid eye movement sleep and ketamine, which are characterized by increased cholinergic tone and EEG activation (Lydic and Baghdoyan, 2005; Abásolo et al., 2015; Pal et al., 2015; Sarasso et al., 2015; Schartner et al., 2017a). Therefore, it is possible that underlying changes in cholinergic tone are more closely associated with the complexity changes rather than behavioral arousal.

Interestingly, despite the increase in cortical acetylcholine levels or the behavioral phenotype following carbachol or NA delivery into prefrontal and parietal cortices (Pal et al., 2018), the functional connectivity, as measured in the current study, did not show a significant change. Therefore, functional connectivity is doubly dissociated from both behavior and cortical cholinergic tone. Furthermore, our results are consistent with the effect of anesthetics on local neuronal networks. A dissociation between cortical neuronal interactions and spontaneous behavior was demonstrated after stimulation of the ascending arousal system in rats anesthetized with desflurane at comparable effective levels to that used here (Pillay et al., 2014). Notably, previous studies targeting multiple subcortical pathways with a variety of stimulus modalities (electrical, pharmacological, optogenetic) have demonstrated restoration of wakefulness from unconsciousness induced by different classes of anesthetics (Alkire et al., 2007, 2009; Solt et al., 2011, 2014; Chemali et al., 2012; Muindi et al., 2016; Taylor et al., 2016; Gao et al., 2019), suggesting that site of stimulation, rather than stimulus modality or anesthetic agent used for inducing unconsciousness, may play a greater role in modulating behavioral arousal. However, these studies did not investigate changes in connectivity or complexity; therefore, it remains to be seen whether the restoration of wakefulness from anesthesia after subcortical stimulation will produce connectivity and complexity patterns similar to those observed in our study.

Although previous reports of the dissociation between EEG and behavioral states (Maksimow et al., 2006; Hobson, 2009; Qiu et al., 2015; Gaskell et al., 2017; Sidorov et al., 2017; Sanders et al., 2018b) are supportive of our results, caution should be applied in the interpretation of these findings because of the reliance of our study on behavioral motor activity for the determination of wakefulness, the inherent limitations of the connectivity and complexity measures used, and the inability to make any inferences related to the content or experiential nature of conscious activity. Furthermore, although we have artificially dissociated levels of consciousness and various cortical measures, it is still possible that they correlate during more typical physiological and pharmacological state transitions or with other techniques of neurophysiology, functional neuroimaging, and analysis.

In conclusion, we conducted a systematic comparison of multiple EEG measures (connectivity, complexity, and spectral power) after cholinergic or noradrenergic stimulation of distinct cortical areas, and demonstrate the dissociation between these cortical measures and level of consciousness.
